# A field test of empathetic refutational and motivational interviewing to address vaccine hesitancy among patients

**DOI:** 10.1038/s41541-025-01197-8

**Published:** 2025-07-03

**Authors:** Angelo Fasce, Mirela Mustață, Alexandra Deliu, Dawn Holford, Linda Karlsson, Virginia Gould, Gheorghe Gindrovel Dumitra, Dana Farcasanu, Iulia Vișinescu, Pierre Verger, Stephan Lewandowsky

**Affiliations:** 1 University of Erfurt, Erfurt, Germany; 2 Southeastern Health Regional Observatory, Marseille, France; 3 Center for Health Policies and Services, Bucharest, Romania; 4 Romanian Academy, Bucharest, Romania; 5 University of Bristol, Bristol, UK; 6 University of Turku, Turku, Finland; 7 University of Medicine and Pharmacy of Craiova, Craiova, Romania; 8 Romanian National Society of Family Medicine, Bucharest, Romania; 9 University of Potsdam, Potsdam, Germany

**Keywords:** Vaccines, Public health

## Abstract

Vaccine hesitancy is among the most concerning public health issues due to declining immunization rates worldwide. We report a mixed-methods field test of two conversational techniques that allow for an empathetic dialogue on vaccination between health care professionals and patients: Empathetic-refutational interviewing (ERI) and motivational interviewing (MI). Thirty Romanian general practitioners were assigned to an untrained control group and to two experimental groups in which they were trained in ERI or MI. After training, physicians had conversations on HPV and influenza vaccines with 334 patients who were hesitant to receive a vaccination. Patients of physicians in the ERI group demonstrated larger increases in positive attitudes toward vaccines and willingness to get vaccinated, while a greater proportion of patients in the MI group scheduled vaccination appointments. Interviews with participating physicians revealed overall satisfaction with the conversational techniques. Empathetic interpersonal communication can have a substantial positive impact on vaccination rates, especially for vaccines subject to mass misinformation campaigns.

## Introduction

Despite the resounding success of immunization campaigns worldwide, vaccines have a long history of encountering patients’ hesitancy (i.e., delay in acceptance or refusal of safe vaccines despite availability of vaccination services), which the World Health Organization has identified as one of the greatest threats to public health^1^. The COVID-19 pandemic and the associated emergence of misinformation around vaccination gave rise to an environment conducive to the proliferation of antivaccination beliefs^2^.

This context of misinformation around vaccinations makes it necessary that communication interventions for the promotion of health decisions are developed and implemented based on the best available scientific evidence^3^. Even though such interventions have been developed within the framework of mass communication, especially for social and mass media, they are still insufficient for face-to-face interactions between health care professionals and patients—a setting which is crucial for dealing with vaccine hesitancy^4^. Despite the potential for health care professionals to directly debunk misconceptions surrounding vaccines^5^, prior evidence suggests that rebutting misinformation is difficult and not always successful—especially if the rebuttal ignores people’s deeply-held beliefs and psychological predispositions^6^. For that reason, a personalized communication approach is more likely to successfully correct erroneous beliefs than non-personalized communication of scientific evidence, especially if the personalized approach considers the diversity of underlying psychological motives that drive hesitant attitudes toward vaccines. However, given the difficulties expressed by physicians in addressing vaccine hesitancy^7^, the implementation of communication interventions requires specific training programs for health care professionals.

In this study, we conducted a mixed-methods field test of two communication approaches for health care professionals that, although related in some of their conceptual foundations, pursue different approaches to tackle vaccine hesitancy among patients: the recently developed empathetic-refutational interview (ERI) and the motivational interview (MI)^8^. The objective was to assess the impact of the ERI and MI on the following outcome measures: attitudes toward vaccines, willingness to get vaccinated, appointments to get vaccinated, satisfaction with the interaction with the physician, and whether the patient still had doubts about vaccination after the conversation with the physician. These two programs were implemented in Romania, a country that presents particular challenges with vaccine hesitancy and has some of the lowest vaccination rates in Europe^9^. Our results illustrate the effectiveness of both communication interventions in naturalistic settings and shed light on strengths and challenges for the implementation of both training programs on a larger scale.

Romania has a mandatory social health insurance system, where the primary health care services are delivered by family physicians’ offices, who serve as the system’s gatekeepers. These general practitioners are self-employed or private providers delivering preventive and curative health services under a contract with the county’s healthcare system, based on a mix of capitation (i.e., a fixed amount of money per patient per month paid in advance to the physician for the delivery of health care services) (35%) and fee for service (65%). General practitioners provide patients with vaccinations free of charge, following the guidelines established by the National Immunization Program, which ensures equitable access without imposing vaccination mandates. General practitioners thus play a pivotal role in the vaccination process by assessing vaccine eligibility, offering guidance, administering vaccines, and monitoring adverse reactions. Their vaccination efforts are complemented by support from other primary healthcare professionals, including nurses from their place of practice and community nurses, who provide health education and promote vaccination, mainly in rural areas.

The immunization landscape in Romania presents a concerning picture^10^. A national survey conducted in 2019 revealed that only 55% of Romanian respondents believed the benefits of vaccination outweighed its risks, while 8% viewed childhood vaccination as unnecessary^11^. This local prevalence of vaccine hesitancy has been fueled by the COVID-19 pandemic, as shown by a declining confidence in childhood vaccines of up to 10 percentage points, with 13.4% fewer Romanians under 35 expressing confidence in vaccines after the pandemic. This decline was more pronounced among men (14.6%) compared to women (5.7%)^10^. Several factors still contribute to the rising threat of vaccine hesitancy in Romania, including uncertainty about the pandemic response, increased access to misleading information, declining trust in experts, and growing political polarization^10^.

The ongoing campaign for the human papillomavirus (HPV) vaccine offers an interesting opportunity to assess how public attitudes shape vaccination efforts. Romania’s initial attempt at an HPV vaccination campaign in 2008 saw limited success, with only 2% of teenage girls receiving the vaccine, partly due to fears that it was part of an experimental drug testing program^12^. The Romanian Government reintroduced the HPV vaccine in the National Immunization Program in 2012, but the vaccine was not available free of charge until January 2020, based on a preorder mechanism when the parents sign a request. In December 2023, a new mechanism to provide the vaccine through electronic prescription directly to the beneficiary was introduced, making it available through family physicians. Currently, the vaccine is free for girls and boys aged 11–18 and is 50% reimbursed for women over 19, marking a renewed effort to boost vaccination rates^13^. However, recent data released by UNICEF indicate that, in 2023, only 6% of eligible girls in Romania were vaccinated against HPV^14^.

In the case of the influenza vaccine, recent flu seasons have shown a decline in uptake, with overall vaccination coverage at just 5.7%, down from 8% the previous season. Among those aged 65 and over, coverage dropped to 19.6%, far below the WHO target of 75%, while healthcare worker vaccination rates plummeted to 5%, compared to 8% in 2022–2023, 21.5% in 2021–2022, and 45.9% in 2020–2021^15^. Within this context, the National Institute of Public Health highlights the need for increased promotion of flu vaccination, in particular efforts to combat vaccine hesitancy through educational campaigns and transparent communication on vaccine safety and effectiveness^16^. The influenza vaccine is fully reimbursed for specific population groups under Government Decision No. 720/2008, which includes children aged 6 months to 19 years, pregnant women, adults aged 19 to 65 with chronic conditions, individuals over 65, and healthcare professionals^17^.

MI constitutes a communication framework originally developed to address substance abuse problems by emphasizing a therapeutic relationship based on the evocation and reinforcement of intrinsic motivation for change^18^. Since then, this approach has become a well-established intervention for a wide range of health problems requiring patient behavioral change, such as dietary habits, weight reduction, or harmful sexual practices^19,20^. The cornerstone of MI is the empowerment of the patient in making behavioral changes and medical decisions through the assistance of the health care professional, who should strive to establish a partnership with the patient^20^. Rather than seeking to persuade the patient, the main goal of MI is to ignite motivation, foster change, and thus support the patient by using a set of core communication skills known as OARS (i.e., open-ended questions, affirmations, reflections, and summaries) and a general communication approach known as CAPE (i.e., compassion, acceptance, partnership, and evocation)^21,22^.

In the context of vaccine hesitancy, MI is based on a 4-step practical framework^21^:

1. Engage: Establish a trustful, judgment-free relationship with the patient and a safe place to talk about vaccines.
2. Understand: Identify “what matters” most to the patient in their hesitant attitude.
3. Offer information: Use ask-offer-ask to provide targeted information that addresses their concerns about vaccines.
4. Clarify and accept: Validate the patient’s autonomy in their decision on getting vaccinated.

The effectiveness of MI in promoting more positive attitudes toward vaccination, including increased intention to vaccinate, has been well documented in previous field tests conducted in the U.S.^23,24^, Canada^25–27^, and France^28^.

Similar to the MI, underlying the ERI is the principle of empathy, conceptualized as communicating understanding of patients’ experiences, concerns, and perspectives^8,29^. However, another fundamental goal of the ERI is to address misconceptions about vaccination by considering the “attitude roots” of anti-vaccination beliefs. Attitude roots are the beliefs, ideologies, fears, and identity issues that motivate people to want to reject a scientific consensus^30,31^. For example, an attitude rooted in a tendency to believe in conspiracy theories may manifest itself in the argument that one should reject “official” medicine because it is part of a secret plot to increase the financial benefits of so-called “Big Pharma”^32,33^.

Understanding these attitude roots allows a refutation to be aligned with the individual’s motivations, thereby avoiding triggering the individual’s defensive posture, which might lead them to reject a corrective message. Therefore, the ERI also addresses misconceptions that patients may hold, while at the same time affirming, to the extent possible, the attitude roots of vaccine misinformation, such as distrust, moral and religious concerns, or pseudoscientific conceptions^30^. To this end, the ERI training program equips healthcare professionals with various strategies to affirm the patient (i.e., acknowledge partial truth, offer praise, accept their perspective, and normalize feelings) and address their arguments against vaccination (i.e., explain misconception, offer an alternative narrative, tailor the refutation to the attitude root, and redirect patient’s perspective), to be implemented in accordance with the patient’s psychological profile (e.g., how strongly opposed to vaccines they are and how central the root attitude is to their personal identity).

The ERI also exhibits a 4-step structure^8^:

1. Eliciting concerns: Open-ended questions and active listening to give the patient freedom to express themselves and to give the healthcare professional an opportunity to identify the patient’s attitude roots.
2. Affirmation: Showing empathy toward the patient’s position by expressing understanding of their concerns and motivations (without, however, endorsing misinformation).
3. Tailored refutation: Explaining why the patient’s misconception is wrong, replacing the misconception with an acceptable alternative explanation without challenging the underlying attitude root.
4. Providing factual information: Presentation of evidence-based information about vaccinations.

The ERI has been developed within the framework of the EU Horizon 2020 JITSUVAX project, which has already gathered initial evidence of its efficacy. In a series of online experiments in which participants evaluated a simulated physician–patient interaction, Holford et al. found that the use of empathetic refutational messages, in comparison to a general refutation and a non-empathetic tailored refutation^8^, led to greater support and perception of the compellingness of a physician’s communication style, and more trust and openness toward the physician among vaccine hesitant participants.

MI and the ERI have two main differences, which are reflected in the skills acquisition of healthcare professionals during their respective training courses. First, the ERI promotes empathetic and direct correction of misconceptions^8^. Refutation is not part of MI, even though in this approach, healthcare professionals offer information to patients after asking permission^21^. Second, a central feature of MI, which is not part of the ERI, is to support behavior change^18,20^. Within the framework of MI, during an interaction with a patient, healthcare professionals should be able to identify and encourage the “change discourse” (i.e., the elements of the patient’s discourse that evoke the importance of vaccination and reinforce confidence in it)^18,21^.

In this field experiment, we assessed the effectiveness of ERI and MI on patients’ vaccination attitudes and behaviors. For this, physicians were allocated to training in either ERI or MI and then had conversations with patients who were hesitant to receive a vaccination. A third group of physicians was allocated to a no-training control group. Comparisons of the patients’ responses (described in the Methods) before and after the conversation with the physician served to evaluate whether ERI and MI have a stronger effect on vaccine-related attitudes and behaviors than the control condition with no training. Furthermore, comparisons of the patients’ evaluation of physicians using ERI and MI served as the evaluation criteria for effects on interpersonal communication. We also measured health care professionals’ perceptions of the training sessions through semi-structured interviews to examine how useful the approaches are perceived by active practitioners.

## Results

### Results of the training among physicians

We conducted a series of paired samples t-tests to evaluate the outcomes of the training sessions in ERI and MI. Table 1 displays the pre-training and post-training comparisons on the International Professionals’ Vaccine Confidence and Behaviors questionnaire (I-Pro-VC-Be), Empathetic Refutational Interviewing Skills in Immunization questionnaire (ERISI), Motivational Interviewing Skills in Immunization questionnaire (MISI), and difficulties in addressing anti-vaccination arguments. The analyses indicate that both training sessions produced significant positive changes in knowledge about the respective technique (and in related behaviors, in the case of MI). However, differences were also observable between the approaches, as only physicians in the ERI group increased their proactive efficacy and confidence in refuting arguments.

**Table 1:** Paired samples *t*-test between the pre-test and the post-test of the training sessions

	ERI				MI			
	Pre-test M (SD)	Post-test M (SD)	*t*	Hedges’ *g*	Pre-test M (SD)	Post-test M (SD)	*t*	Hedges’ *g*
Confidence in vaccines	4.08 (0.33)	4 (0)	−0.709	−0.215	3.95 (0.37)	4.1 (0.32)	1.406	0.426
Proactive efficacy	4.5 (0.24)	4.85 (0.24)	4.583***	1.388	4.35 (0.24)	4.6 (0.32)	2.236	0.677
Trust in authorities	4.8 (0.42)	5 (0)	1.50	0.454	5 (0)	5 (0)	–	–
Openness to patients	4.3 (0.95)	4.7 (0.48)	1.809	0.548	3.6 (1.74)	4.1 (1.20)	1.342	0.406
Perceived constraints	2.50 (1.43)	2 (1.41)	−1.103	−0.334	2.7 (1.7)	2.5 (1.27)	−0.688	−0.208
Reluctant trust	3.11 (1.15)	3.67 (1.66)	−1.644	0.522	3.7 (1.34)	2.9 (1.85)	−1.309	−0.397
Knowledge about the technique	8.7 (2.26)	13.1 (1.29)	6.41***	1.941	4.1 (1.1)	7.1 (1.60)	5.031***	1.524
Behaviors related to the technique	–	–	–	–	3.88 (0.66)	4.64 (0.51)	3.389**	1.026
Difficulties in addressing arguments	2.33 (0.6)	1.45 (0.38)	−3.639**	−1.102	2.53 (0.69)	2.29 (0.92)	−0.89	−0.27

*Note*. Significant differences in bold. *p < 0.05, **p < 0.01, ***p < 0.001.

To further explore intergroup differences between ERI and MI, we conducted independent samples t-tests on the post-training measures of the following variables: confidence in vaccines, proactive efficacy, difficulties in addressing arguments, and perceived competence in the trained technique (see Table 2). The results suggest that the ERI group finished the training with lower levels of perceived difficulty for addressing anti-vaccination arguments and a greater perception of competence in the trained technique compared to the MI group. These results remained stable after controlling for the baseline—there are no significant differences between groups in the pre-test measures.

**Table 2:** Independent samples t-test between the ERI group and the MI group in post-test variables of interest

	ERI M (SD)	MI M (SD)	*t*	Hedges’ *g*
Confidence in vaccines	4.00 (0)	4.10 (0.32)	1.000	0.428
Proactive efficacy	4.85 (0.22)	4.60 (0.32)	−1.987	−0.851
Difficulties in addressing arguments	1.45 (0.38)	2.29 (0.92)	2.647*	1.134
Perceived competence in the technique	9.47 (0.4)	7.90 (1.7)	−2.626*	−1.211

*Note*. Significant differences in bold. *p < 0.05, **p < 0.01, ***p < 0.001.

### Attitudes toward vaccines and willingness to get vaccinated among patients

**Fig. 1:**
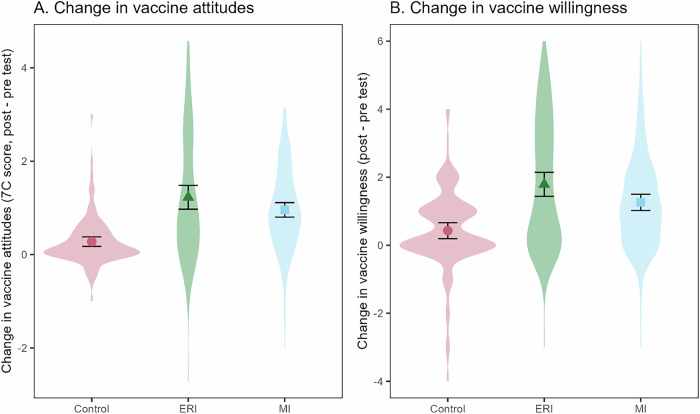
Change in attitudes toward vaccines and willingness to get vaccinated by group. Colored points with error bars show the mean change for each group and their 95% confidence intervals. Violins show the smoothed distribution of the change for each participant in the respective groups. Panel **A** displays change in vaccine attitudes, while panel **B** displays change in vaccine willingness.

We then investigated the effects of the communication approaches (i.e., ERI and MI) on patients’ attitudes towards vaccines (i.e., average response to the 7 C scale) and willingness to get vaccinated. As shown in Fig. 1, the increase in positive vaccine attitudes and willingness was larger in the ERI and MI groups than the control group, with average increase in positive vaccine attitudes of +1.23 (ERI) and +0.96 (MI) compared to control (+0.28), and average increase in vaccine willingness of +1.79 (ERI) and +1.26 (MI) compared to control (+0.43).

We first ran our pre-registered repeated measures analysis of variance (ANOVA) with timing of the measure as the repeated measure and group as a fixed factor. In this model, the critical interaction between timing and group indicates the difference in attitude or vaccine willingness change (i.e., between the pre-test baseline and post-test) between the groups. This critical interaction was significant, indicating a difference in increasing positive vaccine attitudes between ERI, MI, and control groups, *F*(2, 331) = 26.06, *p* < 0.001, *η*^2^_*P*_ = 0.14. This interaction was also significant for vaccine willingness, *F*(2, 327) = 21.11, *p* < 0.001, *η*^2^_*P*_ = 0.11. The full results of the ANOVA (including the main effects) are reported in the Supplementary Material (Supplementary Table 1).

However, this larger observed increase might mask two underlying factors: first, there were baseline differences between groups, with patients in the ERI group holding significantly more negative vaccine attitudes (estimated *M* for control = 5.42, ERI = 3.97, and MI = 4.89) and being significantly less willing to get vaccinated before the consultation than those in the control group (estimated *M* for control = 5.39, ERI = 4.20, and MI = 5.00; see Supplementary Table 2). Second, patients were nested within physicians, creating clustering of observations. We therefore accounted for these two factors in an additional analysis of covariance (ANCOVA) following recommended analytical procedures for imbalanced pre-test measures—i.e., using post-test scores as outcome measure and adjusting for pre-test scores by including it as a predictor together with the group variable^34,35^. Further, to account for the clustering of patients among physicians, we used linear mixed-effects (LME) models for the analysis, which nested patients within physicians. We used the lme4 package (version 1.1-31) in R and modeled random intercepts for physicians^36,37^. The results from these additional analyses are displayed in Table 3 and resulted in no significant effects of communication approach on post-test vaccine attitudes (estimated *M* for control = 5.22, ERI = 5.66, and MI = 5.65). Nevertheless, a significant effect of communication approach on post-test vaccination willingness was found (estimated *M* for control = 5.46, ERI = 6.24, and MI = 6.06), as patients of physicians who had received ERI training were more willing to get vaccinated at post-test compared to the control group.

**Table 3:** Results from LME analyses on patients’ vaccine attitudes and vaccination willingness

Outcome	Fixed effects	*b*	SE	*t*	*p*
Post-test vaccine attitudes	Intercept	−0.28	0.17	1.65	0.111
	Group: ERI	0.38	0.24	1.59	0.123
	Group: MI	0.36	0.24	1.55	0132
	Pre-test vaccine attitudes	**0.80**	**0.04**	**19.28**	**<0.001**
Post-test vaccination willingness	Intercept	**−0.31**	**0.15**	**2.15**	**0.040**
	Group: ERI	**0.50**	**0.21**	**2.40**	**0.023**
	Group: MI	0.38	0.20	1.88	0.070
	Pre-test vaccination willingness	**0.64**	**0.05**	**13.25**	**<0.001**

*Note*. Pre- and post-test variables were *z*-scored. Intercept represents the control group mean. Significant effects in bold.

### Patients’ satisfaction, doubts, and vaccination appointments

**Fig. 2:**
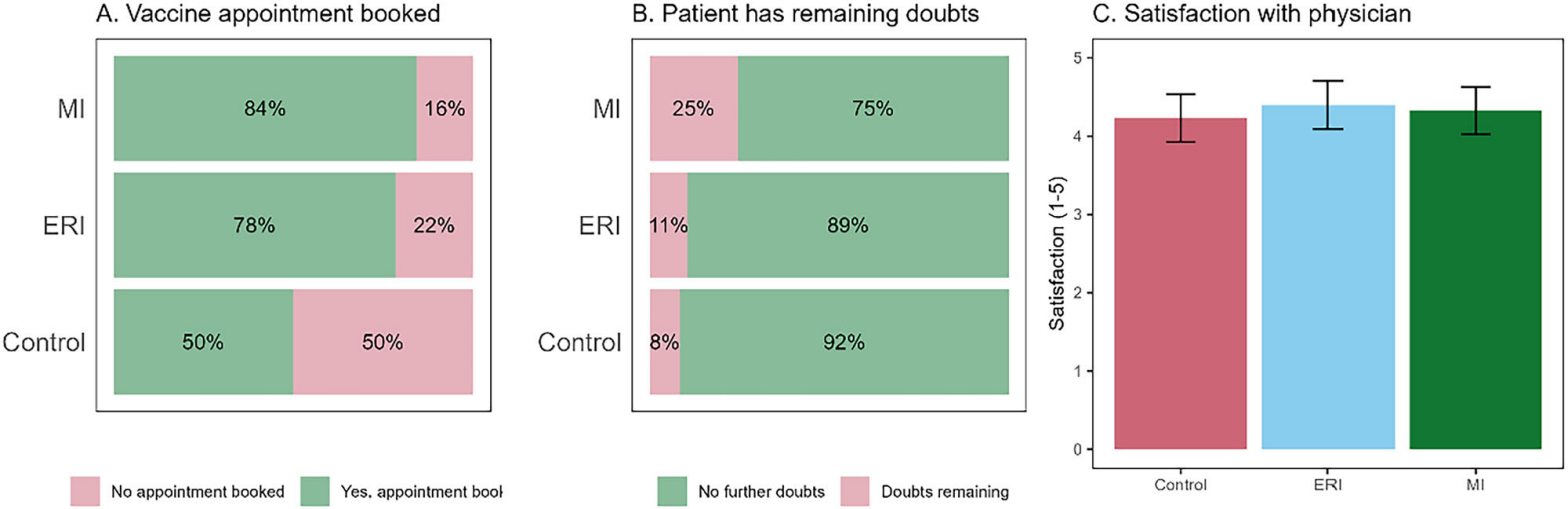
Effects of communication approaches on post-consultation outcomes. Predicted probability of each outcome in the mixed-effect models for vaccine appointment booked (panel **A**) and remaining doubts for patients (panel **B**), and reported satisfaction with physician (panel **C**), by condition. *Note*. Only the difference between the MI and control group in vaccine appointment booked (panel **A**) was significant in the statistical analysis.

Next, we investigated differences between the groups in variables measured only at post-test: patients’ reported satisfaction with their interaction with the physician, whether patients had remaining doubts about vaccination, and whether they booked vaccination appointments. For the satisfaction variable (i.e., patients’ average response to the satisfaction-related items) we conducted an LME analysis, whereas for the doubts and appointment variables that were dichotomous (i.e., yes or no) we carried out generalized linear mixed-effects (GLME) analyses. In all three analyses, group was included as a fixed effect and physician as a random intercept. Because of the previously mention pre-test differences, and the fact that satisfaction, doubts, and appointment bookings were measured only at post-test, we controlled for vaccine attitudes by including the pre-test in the 7 C scale as a fixed factor. These analyses are displayed in Table 4 and Fig. 2.

**Table 4:** Results from LME and GLME analyses on satisfaction, doubts, and appointment bookings

Outcome	Fixed effects	*b*	SE	*t*	*p*
Satisfaction	Group: ERI	0.30	0.31	0.97	0.339
	Group: MI	0.17	0.30	0.56	0.579
	Vaccine attitudes	**0.35**	**0.06**	**5.95**	**<0.001**

Outcome	Fixed effects	*b*	SE	*z*	*p*
Doubts	Group: ERI	0.25	1.08	0.23	0.817
	Group: MI	1.31	0.92	1.42	0.157
	Vaccine attitudes	0.17	0.32	0.53	0.597
Appointments	Group: ERI	2.25	1.21	1.85	0.064
	Group: MI	**2.66**	**1.24**	**2.15**	**0.031**
	Vaccine attitudes	**1.19**	**0.26**	**4.63**	**<0.001**

*Note*. Significant effects in bold.

The analyses showed no significant difference between groups in how satisfied patients were with the consultation or whether they had remaining doubts after the consultation. Nevertheless, the patients of the physicians who had received MI training were more likely than those in the control group to report having booked a vaccination appointment (+34%; the increase was +28% for ERI relative to the control group, although not statistically significant).

### Semi-structured interviews with physicians

At the end of the experiment, we conducted semi-structured interviews with the participating physicians. These interviews were composed of five fixed questions, with the possibility of following up if the physician raised an interesting issue. Interviews were conducted by telephone over approximately 3 months in 2024, with the first interview being conducted on February 21st and the last one on June 4th. The resulting transcriptions in Romanian were analyzed using NVivo 11, with codes adapted to the experiences of physicians either as part of one of the experimental groups or the control group. In addition to the codes corresponding to the questions in the interview guide, there were also codes depicting topics that appeared throughout the interviews, broadly related to vaccination but not to the main theme of the interviews.

The main results of the interviews are reported below, structured based on the five fixed interview questions. The interview guide, the codebook, and additional, unexpected topics outside the fixed questions (i.e., general attitudes toward vaccination, physician–patient relations, the effects of the COVID-19 pandemic, time constraints, and issues with the system) can be accessed at https://osf.io/rmwvc/.

#### How satisfied were you using the MI/ERI/your current skills discussing vaccination with your patients?

Physicians from the experimental groups were satisfied with the specific method they applied, as the training proved to be effective and contributed (sometimes drastically) to restructuring the way they interact with their patients. Instead of relying exclusively on their scientific authority, physicians learned how to become partners with their patients and share co-constructed values:

*Very much. And what I liked most of all, that never, and I also use this in my everyday life, I will never say again, and I will say: “It’s not true. I’ll tell you how it is”. No. So I will never, I will not contradict them. I always say: “Yes, it’s true”. [..]come on, look, there is another side, here is how I see the problem. If you ever need to talk, tell me and I’ll help you with what, what I can, you probably also have some slightly erroneous information. And if the person sees that you’re not imposing something on him and it’s not, he’s not [reflexive], I say that it will be a good thing. I found it very, very helpful*. (MI group)

Another source of satisfaction is based on the fact that discussions led to the creation of a safe space for the patients, as their fears and worries were addressed one by one by the physician, whose empathetic positioning went beyond vaccination counseling and became a general approach to social interactions:

*Yes, it helped me a lot, I mean, following those steps for counter-argumentation, basically it was a, a longer interview, a longer discussion, following those steps. And the patient was much safer that way, safe and happy in the end to have been given all this, and the good feedback and the right information afterwards. Okay, he did get them before too. Yes, following these steps, by the book, as in the interview, how we were supposed to proceed, I saw that he was more satisfied. It was better*. (ERI group)

Similar to the opinions expressed by interviewees from the MI and ERI groups, physicians from the control group also displayed significant levels of satisfaction, this time not prompted by a specific method used in the discussions with patients, but by the discussions themselves—even though this could also be due the lower levels of vaccine hesitancy reported for the control group. Even in cases of refusal, physicians mentioned the importance of patients obtaining the information they need:

*I am very satisfied because I understand everyone, and I can say that I am satisfied with the way the discussion is going. Even if they don’t accept, they at least get some advice, and they get some information, after all. They think about it at home*. (Control group)

#### What kind of difficulties and advantages did you encounter?

When asked about the difficulties they encountered while using MI/ERI or while counseling patients for vaccination, interviewees’ first response usually referred to the patients they could not convince to get vaccinated. Some physicians report that they did not encounter patients that totally refused any kind of conversation, but still mention that there were people who stuck to their initial opinions or uncertainties. In these cases, physicians tend to equate their investment in terms of time and emotional work with a loss, and it becomes a source of dissatisfaction:

*Difficulties? Patients who don’t want to discuss, no, that’s not, that didn’t happen. However, as I told you, following the discussion, at some point they showed signs that they understood that it would really be good to do it. But it remained at this stage. That’s the tragedy. And that gives you, I don’t know, the dissatisfaction of your work, after all. That it is a time that you allocate outside of other things, and the result is not up to expectations. That’s not, it doesn’t thrill you*. (MI group)

What also came up in the interviews was the fact that sometimes the arguments invoked by patients were hard to counter because they were unrelated to science or vaccines themselves:

*Sometimes I ran out of arguments, so to speak, because they brought some non-arguments in favor of their opinion, like “I read on the Internet”, “That’s what a neighbor told me”*. (MI group)

*We had, if we’re doing full disclosure, or with utmost honesty, we, right after we finished the course, until we started documenting the interviews, we had a few meetings with patients where we tried to apply the principles, and there have also been moments when the patients, following the discussion, did not gain anything, as a pro-vaccination experience. But, well, we have to accept at some point a percentage of failure, because it is impossible to convince everyone, or to make the same method work for everyone, and then… But the difficulties most of the time have been in terms of the actual, factual information, not necessarily the way it was delivered, or the technique of the interview or interaction*. (ERI group)

Accounts of difficulties from the control group were defined along the same lines, related to patients who are still refusing vaccination even after counseling, with the same observation that the majority of patients were rather receptive and open to conversation.

Regarding advantages, physicians got familiarized with ways to be empathetic with patients and lead discussions, which allowed patients to ask questions and to manifest their fear, ultimately leading to a provision of counseling with a high degree of individualization:

*I mean, the patient is used to being treated rather hastily, like, they are not allowed to ask questions, and this is the first advantage. At such a consultation all 10 were very satisfied simply that it was carried out in this way. I mean, they had a doctor in front of them who seemed interested in all their problems, in all their questions, he [the doctor] didn’t rush them, he didn’t tell them that it’s not done this way and that way, yes, that seems to me to be a huge advantage, for the doctor and for the patient as well*. (MI group)

*One of the advantages was that by trying to start from an understanding of what they think and how they feel about that subject, it’s much, much, much easier for you to, to correct them, let’s say, or direct them, or advise them on their choice, in a personalized way, so to speak. Because not everyone has the same reluctance when it comes to, not only vaccination, but medical procedures in general. And what we think would upset the patient most of the time is not the case*. (ERI group)

Similar views were manifested by physicians in the control group, as discussing with patients motivated them to be more open and made the patients themselves more flexible:

*I think they appreciated and liked the fact that I was open and… Because I haven’t had time, frankly, since I took over the practice, to talk to them about things like that. And the fact that I came and I was open and friendly, so to speak, I mean it was a discussion after all, not necessarily just patient-doctor. I tried to be like a friend, so to speak*. (Control group)

#### How confident and comfortable were you when applying MI/ERI/your current skills discussing vaccination with your patients?

Even though using MI and ERI was described as something totally new by most of the physicians, they also expressed having felt confident after the training:

*(…) the motivational interview as we discussed it in this course, is not something that I have done before. It’s something new for me. No, I have never used to beg a patient this way, or to explain it to a patient in this way*. (MI group)

*I felt very confident and happy that I had, it was discovered, I was shown to me this way, to be able to understand the patient better, to be able to be more attentive to his complaints and to everything, that is, to have more, the be more targeted towards the patient, not only towards, as we are currently forced to have a lot of papers and…* (MI group)

However, interactions with patients holding anti-vaccination beliefs were described as uncomfortable by physicians of all groups. This discomfort seems to be rooted in physicians’ adherence to a certain interactional logic, dominated by the imbalance of power between health care professionals and lay persons. These situations were described as especially frustrating when it comes to dealing with teenagers:

*Some of them irritated me. But that was, how can I tell you, when they say: ‘Ah, I will think about it’. But what are you thinking about? Well, they say: ‘I haven’t started my sex life’. Or: ‘Oh, well, I’ve known my husband for a long time, there’s no need’. You know, the 20 year olds. Or the children: ‘Oh, well, but I don’t know if I need it.’ The reply: ‘I’m thinking about it’. I mean, what are you thinking about if you don’t know anything about anything?* (ERI group)

References to professional experiences on communication and public speaking were recurrent in the interviews. Physicians showed a tendency to compare MI and ERI to sales techniques, and as resources for physicians to “manipulate” the patients, to steer them in the right direction. Physicians also stressed that MI and, especially, ERI, require a certain level of background knowledge and cognitive reflection on the part of patients:

*Well, I have also chosen who to talk to, to have something to discuss with them. Because we keep coming back to this matter, because if he [the patient] only knows how to mix sand with cement, it’s harder to get him to an element of this, for him to understand what happens through immunization*. (ERI group)

The scarcity of reasons for discomfort was also the case for physicians in the control group. There were cases in which interviewees mentioned that they are used to being more outspoken and stricter with their patients, and that they do not spend a lot of time with those who are obviously against vaccination. At the same time, there were also micro narratives centered on how physicians must not take patients’ reactions personally. In this regard, what seemed to be important was to be able to “read” patients and distinguish between the undecided ones, the ones that could still be convinced to get vaccinated, and the patients who have their minds set against vaccines, who should be left alone, as discussions with them are a waste of time:

*(…) that’s the biggest problem, when you take everything personally. And you take it like: “well, so you don’t trust me, what am I saying?” And don’t try, to those who are totally against, with the conspiracy theory, don’t try, because you have no chance. Then, you know this, try with the undecided ones. There. You can talk there. But otherwise, I avoid frustrations*. (Control group)

#### How did the patients react to MI/ERI/your current skills when discussing vaccination?

As for the reactions of patients, the opinions of interviewees were unanimous: patients were usually eager to find out more, and pleasantly surprised by the fact that physicians took some more time than usual to talk to them. In a few cases, the interviewees mentioned that patients were influenced by having to complete questionnaires, which made them more attentive and provoked feelings of uncertainty about how things will evolve:

*Given that they had completed a questionnaire before, they didn’t quite know what to expect. They thought it was something much more elaborate, I don’t know exactly what they expected, and they were like that, kind of scared at first*. (MI group)

Because of traditional understandings of the physician-patient relationship and the severe lack of time experienced by the physicians, the normalcy of the interactions was rather based on things being swiftly discussed. Some interviewees from the experimental groups mentioned that they have never approached the interactions with patients in such an empathetic manner, which was surprising not only to the patients but to themselves:

*I haven’t had the attitude from the course until now at my practice. My change of attitude somewhat surprises them. To see that I talk to them differently, to see that I ask them questions and expect answers from them. To see that I want to discuss and have a longer dialog than the consultation usually lasts. And yes, the vast majority of them were surprised. Receptive, some of them I prayed to them to be able to have discussions, others, others developed it themselves, without me having to do anything*. (MI group)

*Willing to communicate and ask a lot of questions. Yes, they were asking all the questions they had read, heard, yes, yes. That is, it was no longer just their fear. Let’s say they were afraid of something. “No, but I also heard this, but I also heard that…” Yes, they were eager, yes, to know more, especially seeing that I had time*. (ERI group)

Even for the physicians from the control group, the situation was new due to the unusual amount of time allocated to the conversation with the patient. They reported their patients as being delighted to be heard and treated as partners in conversation, and emphasized the need to develop such interactions with patients as a fundamental part of prevention strategies:

*I think most of them were happy. There was also a bit of, a bit of embarrassment in the way, the discussions are quite laborious, that is, you enter a little more, you talk more than you are used to. But I find that people are eager to speak, I mean, it is useful. All in all, I say they are, they would be keen on such a thing. So, if they ever get to do this part of prevention at its true value and these discussions at their true value, I think the population will be at a win*. (Control group)

#### How do you think MI/ERI/your current skills to discuss vaccination with your patients can be improved to better fit the needs of patients and health care professionals?

This final question related to potential improvements to either MI or ERI turned out to be unclear, judging from the first reactions of the interviewees, who tended to refer to the training sessions rather than the method itself. This could be associated with the novelty of the approaches, with trainees not having an extensive knowledge or perception of their own skills that would allow them to reflect on how these methods can be improved to better fit patients’ needs. Most of the interviewees from the experimental groups mentioned that they would like to go further with their personal development and with learning more about MI or ERI. Structural improvements were not an issue in any interview. What was sometimes mentioned was the necessity to adapt MI and ERI to instances of interaction to account for the diversity of possible situations and patient-related specificities:

*It must be adapted to each area, each practice, each doctor and each patient. That’s the improvement you can bring. Because one thing is to talk to people from big cities, university centers, where there is plenty of information, it is much easier. The density of both the population and the information is much higher, and you automatically get a lot more information and something stays in your head in the end*. (ERI group)

*Unfortunately, I don’t think I could tell you. I haven’t had, say, hundreds of patients that I’ve interacted with, to be able to say, yes, here, this thing could be improved, and if there were situations that I could improve on, it’s clear that there are aspects which you improve punctually. I mean it couldn’t, I don’t know if it could be applied on a large scale. Yes, yes, that is, there would be things specific to the respective discussion, which I think everyone can adapt*. (ERI group)

Most of the interviewees from the control group mentioned that training is always needed, especially when it comes to better, more effective ways of communication. The excerpt below illustrates such an opinion, and it brings together the needs of patients to be better informed and to have access to informational materials in a way that is very close to what ERI and MI offer:

*Yes, I think that would be useful. I think it would be useful for us too, as doctors. I don’t know, informational materials, but no, not strictly on the pathology side, because they read that one, they find it, it’s ok. Regarding the interaction with them I think it would help. I mean, I don’t know, certain steps in approaching the discussion, in leading it… In, that’s what I’m thinking, like, for me, as a doctor. And for them, I am convinced that any way to raise awareness is useful. Be it on paper, or, well, less on the way of emails, messages, you know how it is, in the countryside it’s more complicated…* (Control group)

## Discussion

The objective of the reported field test was to assess the impact of two different communication approaches focused on the HPV and influenza vaccines, the ERI and MI, on patients’ attitudes toward vaccines, willingness to get vaccinated, appointments to get vaccinated, satisfaction with their interactions with the physician, and doubts about vaccination. In this regard, the results obtained with the ERI are particularly illuminating as there have been no previous field examinations of its effectiveness. The training of physicians led to good results in both groups, comparable for MI to previous studies in France, Quebec, and the US^38,39^. Both groups increased their knowledge about the communication approaches during training, although interesting differences emerged among the outcome measures, with physicians assigned to the ERI training exhibiting lower levels of perceived difficulty with addressing anti-vaccination arguments and a greater perception of competence in the trained technique compared to their counterparts in the MI group. Moreover, the ERI training produced a greater increase in proactive efficacy (i.e., commitment to vaccination and self-efficacy) than the MI training.

Positive effects among patients were also observed in the field test, with increases in positive vaccine attitudes and willingness for the ERI and MI groups. The changes in these outcomes were significantly greater relative to the control. The significant increase in vaccine willingness for the ERI group remained significant when controlling for pre-test and taking into account the impact an individual physician might have had, indicating the ERI’s promise as a technique to address vaccine hesitancy in face-to-face interactions between patients and physicians. Moreover, despite the refutational nature of the ERI, no differences were observed in patients’ satisfaction with the interaction with the physician. Vaccination appointments, a crucial behavioral outcome that gets closer than the rest of the outcome variables to increasing the uptake of vaccination, made post-conversation was also higher in the ERI (+28%, although not statistically significant) and MI (+34%) groups compared to the control group. Even though an increase in vaccination appointments constitutes a useful proxy for associated increases in vaccination rates, a range of interventions is required for appointments to be successful. In particular, the use of reminders and nudging strategies, such as pre-scheduled appointments to discuss vaccination, has been found to be especially effective in previous research^40,41^.

Across five fixed questions and a wide variety of spontaneous themes, the semi-structured interviews with physicians offered a very positive overview of both the training and the application of MI and ERI in the context of the HPV and influenza vaccines. Physicians in the three groups consistently expressed their satisfaction with the creation of space for empathetic communication with their patients, but the training had a transformational effect on their approach to dealing with hesitant patients, who showed openness and willingness to engage in conversations about vaccination. However, the literature indicates that isolated training would not enable the learning acquired to be integrated into everyday practice^42^, as the addition of feedback and supervision is necessary to foster the acquisition of the ability to use MI^43^. Training health professionals in MI and ERI, therefore, represents a significant investment of time, which may not be compatible with their availability, and represents a challenge to the training of a large number of professionals. In this regard, in the EMMIE program implemented in Quebec to propose an MI to all the mothers of newborns, the Ministry of Health opted to train vaccination advisers rather than physicians^44^. Considering our results, direct training of physicians would benefit communication with vaccine-hesitant patients if supervision is possible and if students have the opportunity to apply the technique with patients.

The obtained results were largely similar in both experimental groups, but more pronounced for attitudes in the case of ERI and for behavioral variables in the case of MI. Even though there is a substantive difference between MI and ERI on the theoretical level (e.g., the ERI requires from the physician a more in-depth analysis and understanding of patient’s speech and the ability to classify the attitude roots to allow for tailored affirmation and refutation of misconceptions), these differences might not automatically translate into differences in implementation of medical practice. Indeed, interviews with physicians assigned to the experimental groups suggest numerous similarities in how situations were defined and evaluated—e.g., physicians’ feelings of comfort/discomfort and patients’ reactions. This might be indicative of empathetic interactions beyond the traditional power relation between physicians and patients playing a crucial role in the observed improvement of the tested outcome variables. Positive effects of taking the time to interact empathetically with patients and listen to their concerns also emerged in interviews with physicians from the control group, albeit very difficult in day-to-day practice due to the lack of time, the high number of patients, and the administrative tasks physicians must take care of. As interviewees in this study did not specify fine details of how they practiced MI and ERI, it would be good for further qualitative research to investigate the practical differences and commonalities between both techniques, particularly to ascertain what physicians tend to put into practice after receiving the respective training.

Despite the strengths of this field test (i.e., high-quality training, proper statistical power, a cultural context of high vaccine hesitancy, post-hoc semi-structured interviews, etc.), it is important to note some of the limitations of our results. First, the effects of the ERI and MI on patients’ vaccination willingness might vary between vaccines, as a result of what attitude roots are commonly associated with hesitancy toward the vaccines. Most of the counseling sessions were in relation to the HPV vaccine, which might be less subject to attitude roots such as conspiracy ideation, which is especially resistant to change^45^. However, some of the attitude roots typically involved in hesitant attitudes toward the HPV vaccine are also difficult to address in a conversation—e.g., a perception of the vaccine as unnecessary because it addresses an illness associated with active sexual behaviors and, often implicitly, the existence of multiple partners, which often constitute complicated topics, especially in communities that are more traditional and adherent to specific gender roles^46^.

Second, due to the inherent characteristics of this field test (i.e., physicians selecting their respective group, and practicing in medical facilities distant from each other and with patients already registered with them within the Romanian health care system), it was not possible to achieve matched randomization across the groups at the baseline of the experiment: each group started from different levels of vaccine hesitancy. Even though physicians were given the opportunity to select their group based solely on their time availability to attend the training session (i.e., not based on information about the contents of the training), which substantially reduces the risk of self-selection bias, we cannot rule out this possibility. It is thus possible that some of the observed increases in positive attitudes toward vaccines and vaccination willingness can be attributed to unexpected self-selection bias or regression to the mean, especially in the ERI group. Nevertheless, the increases in these outcome variables were significantly larger in the ERI group compared to the control group, even when adjusting for the baseline imbalances, suggesting that the ERI approach produced positive changes beyond regression-to-the-mean effects. Furthermore, the increase in attitudes toward vaccines and vaccination willingness in the ERI group can be seen as remarkable when considering the fact that the group was the most vaccine-hesitant at baseline, making them a particularly challenging target for change.

Third, based on the semi-structured interviews, physicians in the control group seemed to have interacted with their patients more than usual, which might have increased the effect size of the pre/post post-comparison of the control group. The control condition in this research involved no training, which allowed physicians to implement their own communication style. This choice represents a “care-as-usual” control condition, thus giving rise to variability in how physicians communicate. 50% of patients in the control group scheduled a vaccine appointment following consultation, which seems remarkably high for a group of vaccine-hesitant patients. It would be valuable for future research to assess the potential positive effects of physicians having a dedicated conversation with vaccine-hesitant patients without any special training. However, an analysis controlling for the effects physicians themselves may have had regardless of training indicated that the changes were indeed greater in the experimental groups. For further standardization of the intervention, it would also be of interest for future research endeavors to compare the ERI to a style of communication that focuses only on facts (i.e., no-empathetic refutation), in an experimental design similar to that used in this study. Non-empathetic refutation has shown promising effects on belief change despite patients’ lower satisfaction with the interaction with the physician^7^.

Our mixed-methods results obtained in Romania under naturalistic conditions (i.e., conversations in real medical consultations) suggested that the use of empathetic conversational strategies may be effective to address vaccine hesitancy in face-to-face conversations between physicians and patients. Both MI and ERI (in this case, applied to the context of the HPV and influenza vaccines) showed significant effectiveness compared to the control group, with ERI showing slightly superior results to MI in two key outcome variables: attitudes toward vaccines and willingness to get vaccinated. Moreover, semi-structured interviews revealed broad satisfaction among physicians and patients with the training and implementation of the techniques under assessment. More research is needed to address potential implementation constraints, such as time availability during consultations and long-term effects. However, these results should encourage the development of strategies for large-scale implementation of both techniques, especially in countries with high levels of vaccine hesitancy, such as Romania.

## Methods

### Ethics and open science

This field test received approval from the ethics committee of the Romanian Center for Health Policies and Services. Informed consent was obtained from all participating physicians and patients.

The experimental design, variables, recruitment strategy, inclusion criteria, and sample size calculations were pre-registered at https://osf.io/p87m3. We expected to observe larger improvements between pre-test and post-test measures of the dependent variables in both the MI and ERI groups in comparison to the control group (i.e., more positive attitudes towards vaccines and more willingness to get vaccinated). Comparisons between the MI and ERI groups in post-test measures of patients’ satisfaction, doubts, and vaccination appointments were deemed exploratory.

All data, questionnaires, and codes used in the study are available at https://osf.io/rmwvc/.

### Recruitment and training of health care professionals

The field test took place in Dolj County, located in the southwest of Romania, with 599,567 inhabitants—the 7th largest county in terms of population size^47^. The recruitment of physicians for this study was conducted through an open announcement disseminated via the discussion lists of the National Society of General and Family Medicine, Dolj branch, which includes 390 physicians. The announcement invited pairs of experienced physicians with current vaccination responsibilities and nurses (with a role in patients’ data collection) to participate in the intervention, without targeting specific individuals or practices. Physicians who expressed interest in the study were given the opportunity to choose one of three participation pathways: (1) inclusion in the control group, which involved participation without prior training, (2) inclusion in the ERI group, which required attending a training session on Saturday, January 27th, 2024, or (3) inclusion in the MI group, which required attending a training session on Sunday, January 28th, 2024. Following the initial expression of interest, participation lists were created for each group. Physicians were subsequently contacted individually, in the order in which they had registered, and the study conditions were explained in detail. In the control group, the first 10 physicians who agreed to participate were selected, while 10 physicians declined participation or were not contacted. In the ERI group, 10 physicians agreed to participate, with 5 declining participation or not being contacted. In the MI group, 10 physicians agreed to participate, while 11 declined participation or were not contacted.

Based on the amount of time required to complete their respective tasks (i.e., training, consultations, and data gathering), physicians in the experimental group were offered €250 in exchange for their participation, physicians in the control group were offered €200, and the nurses were offered €100 for their contribution. The initial expectations, based on our sample size calculations, of having 10 physicians per group were met (i.e., 10 trained in ERI, 10 trained in MI, and 10 who did not receive training for the control group), with a total of 30 nurses who collected data from patients. Sociodemographic characteristics of participating physicians can be found in the Supplementary Material (Supplementary Table 3).

The training sessions for the physicians assigned to the experimental groups were facilitated by M.M., who was previously trained in ERI by a member of the EU Horizon 2020 JITSUVAX project (A.F.), with the support of I.V., a research assistant, and G.G.D., a local general practitioner who also serves as a university lecturer. Each 6-hour training session (one for ERI and one for MI) took place within the facilities of the University of Medicine and Pharmacy of Craiova.

The ERI training included a theoretical module and several role-playing exercises that helped physicians understand the conceptual foundations of ERI and acquire the necessary skills to apply the ERI in conversations with patients. Prior to the training, a web tool displaying comprehensive information about the attitude roots, affirmations, and empathetic refutations used in ERI was translated into Romanian (https://jitsuvax.info/ro/). Physicians used this tool during training, and its subsequent use was encouraged. The MI training was built upon training materials produced by the Romanian Center for Health Policies and Services in collaboration with Arnaud Gagneur (University of Sherbrooke), which were previously used in a similar training program for physicians^26,48^. As with ERI, the MI training comprised both theoretical modules and practical role-playing exercises to provide physicians with a deep understanding of the conceptual foundations of MI and equip them with the requisite skills to effectively implement the intervention. Moreover, concise guides were developed for each vaccine covered in the training (i.e., influenza and HPV), emphasizing key messages and addressing common misconceptions. These guides were provided as tools to all participants in both ERI and MI training to support informed communication.

### Sample of patients

Immediately after completing the training, physicians applied the acquired skills in conversations focused on the vaccines against HPV or influenza with a minimum of 10 patients who were identified as hesitant (data collected between February 12 and May 16, 2024). The first 10 patients from each physician who made an appointment as part of routine health care and were eligible for one of the vaccines (i.e., influenza and HPV), but were not willing or hesitant to receive the vaccination, were asked to take part in the study. Those who declined to participate were replaced by the next ones matching the inclusion criteria. The timing and place of the additional consultation focused on discussing vaccination were determined by the physician, either to align with the patients’ preferences or to ensure an appropriate context for addressing vaccine hesitancy effectively. Patients’ questionnaires were administered before and after this second consultation by the nurses involved in the study. The proportion of conversations dedicated to HPV and influenza vaccination varied according to the specific needs and priorities of each patient population, which differed across practices. However, interviews conducted with physicians following their activities indicated that HPV vaccination was more frequently addressed because the study was conducted outside the flu season. While specific numbers are provided in only a few of the semi-structured interviews with the participating physicians, an approximate proportion of HPV consultations is at least 70% of the total.

Sample size calculations using G*Power (v.3.1.9.7) for the group comparisons in the main outcome variables were performed to estimate a minimal desirable size of patients of 252 for medium effect sizes of 0.22 with 95% power (*α* = 0.05). This minimum sample size was exceeded in the study, with a final sample of 334 patients: 105 in the ERI group, 127 in the MI group, and 102 in the control group.

### Measures

To evaluate the acquisition of ERI and MI-specific skills, the physicians completed shortened versions of the MISI and the ERISI at the beginning of the training and again at its end^49^. The responses of both questionnaires were grouped into two overarching variables: Knowledge about the technique (e.g., identification of attitude roots), and, in the case of MI, behaviors related to the technique (e.g., if they prefer to use questions that call for limited development or elaboration). Moreover, the post-test of both MISI and ERISI included a section on perceived competence in the trained technique. Both groups of physicians also completed a scale on perceived difficulties in addressing vaccination counterarguments with patients, extracted from (e.g., “vaccines overwhelm the immune system, especially when taken in many doses”)^32^. Lastly, to measure the impact of the training courses on the determinants of vaccination behavior among health care professionals, physicians completed the short version of the I-Pro-VC-Be^50^.

Following previous work by Verger et al., the items of the I-Pro-VC-Be were grouped into six constructs^51^: Confidence in vaccines, composed of items reflecting perceived risks of vaccines (i.e., how safe physicians perceive certain vaccines to be), complacency (i.e., the perception of lack of usefulness of vaccines), perceived benefit-risk balance of vaccines (i.e., the degree to which physicians perceive that the benefits of vaccines outweigh their potential risks), and perceived collective responsibility (i.e., the extent to which physicians recommend vaccines to contribute to community immunity); trust in authorities (i.e., trust in institutions and health authorities to provide reliable vaccine information and to define the vaccination strategy); perceived constraints (i.e., perceived practical constraints, such as cost of or access to vaccines); proactive efficacy, composed of items reflecting commitment to vaccination (i.e., the extent to which physicians are proactive in motivating their patients to accept vaccinations) and self-efficacy (i.e., how prepared physicians feel in terms of knowledge and skills to address vaccination with patients); reluctant trust (i.e., the “leap of faith” to trust vaccines and policies even if physicians have doubts); and openness to patients (i.e., positive attitudes toward hesitant patients).

The effectiveness of training in MI or ERI was assessed by examining the outcomes of physician-patient interactions in the context of a conversation about vaccination. The pre-consultation questionnaire for patients included a Romanian version of the 7 C scale for vaccination readiness (including a set of components that increase or decrease an individual’s likelihood of getting vaccinated, such as confidence, practical constraints, and collective responsibility) and initial willingness to get vaccinated^52^. The post-consultation questionnaire included, besides the 7 C scale and the same question on willingness to get vaccinated, a question asking if the patient scheduled an appointment to get vaccinated, five questions to assess satisfaction with the interaction with the physician, and a question asking if the patient still had doubts about vaccination after the conversation with the physician. We calculated total averages for the items of the 7 C scale in the pre-test and the post-test, which resulted in optimal internal consistencies (0.88 and 0.82, respectively).

Means, standard deviations, response rates, and internal consistencies of the variables can be found in the Supplementary Material (Supplementary Tables 4–7).

## Supplementary information

Supplementary material

## Data Availability

The datasets generated and analyzed during the current study are available in the OSF repository at https://osf.io/rmwvc/.

## Code availability

The underlying code for this study is available on OSF and can be accessed via this link https://osf.io/rmwvc/.
