# Supplementary material for: A field test of empathetic refutational and motivational interviewing to address vaccine hesitancy among patients
Source: NPJ Vaccines. 2025 Jul 3;10:142. doi: 10.1038/s41541-025-01197-8 (PMC12229542; doi:10.1038/s41541-025-01197-8)
Supplement: Supplementary file 1 — Supplementary material [file 41541_2025_1197_MOESM1_ESM.pdf]

Supplementary material for *A field test of empathetic refutational and motivational interviewing to address vaccine hesitancy among patients*

Angelo Fasce[1,2], Mirela Mustață[3], Alexandra Deliu[4], Dawn Holford[5], Linda Karlsson[6], Ginny Gould[5], Gheorghe Gindrovel Dumitra[7,8], Dana Farcasanu[3], Iulia Vișinescu[3], Pierre Verger[1], & Stephan Lewandowsky[5,9]

1. University of Erfurt, Germany
2. Southeastern Health Regional Observatory, France
3. Center for Health Policies and Services, Romania
4. Romanian Academy, Romania
5. University of Bristol, United Kingdom
6. University of Turku, Finland
7. University of Medicine and Pharmacy of Craiova, Romania
8. Romanian National Society of Family Medicine, Romania
9. University of Potsdam, Potsdam, Germany

Supplementary Table 1
Mean increase in vaccine attitudes and vaccination willingness and full results of pre-registered ANOVA across conditions.

Outcome	Condition	Mean increase (SD)	Main effect of condition	Main effect of time (pre-/post-test)	Interaction effect (condition*time)
Vaccine attitudes	Control	0.28 (0.53)	$F(2, 331) = 21.73$, $p < .001$	$F(1, 331) = 234.17$, $p < .001$	$F(2, 331) = 25.05$, $p < .001$
	ERI	1.23 (1.33)			
	MI	0.96 (0.90)			
Vaccination willingness	Control	0.43 (1.21)	$F(2, 327) = 3.41$, $p = .034$	$F(1, 327) = 194.33$, $p < .001$	$F(2, 327) = 21.11$, $p < .001$
	ERI	1.79 (1.85)			
	MI	1.26 (1.38)			

Supplementary Table 2
Comparisons between groups in patients' pre-test vaccine attitudes and vaccination willingness.

Outcome	Fixed effects	*b*	*SE*	*t*	*p*
Pre-test vaccine attitudes	**Intercept**	**0.51**	**0.20**	**2.53**	**.018**
	**Group: ERI**	**-1.04**	**0.29**	**3.66**	**.001**
	Group: MI	-0.38	0.28	1.33	.194
Pre-test vaccination willingness	Intercept	0.34	0.21	1.63	.115
	Group: ERI	**-0.65**	**0.29**	**2.24**	**.033**
	Group: MI	-0.21	0.29	0.74	.468

Note. Pre-test variables were z-scored. Intercept represents control group mean.

Supplementary Table 3
Physicians' distribution by gender and place of practice across groups.

Group	Man	Woman	Urban	Rural
Control	1	9	5	5
ERI	4	6	8	2
MI	3	7	4	6
Total	8	22	17	13

Supplementary Table 4

Items administered to all the physicians.

Variable	Item	Range	Pre-test M(SD)	N	Post-test M(SD)	N
Confidence in vaccines		1-5	4.01(0.35)	20	4.05(0.22)	20
	Vaccines are safe.	1-5	5(0)	20	5(0)	20
	Today, some vaccines recommended by the National Vaccination Programme are not useful, because the diseases they prevent are not serious.	1-5	1.30(0.98)	20	1.20(0.89)	20
	The benefits of vaccines outweigh their potential risks.	1-5	4.80(0.89)	20	5(0)	20
	I recommend the vaccines on the vaccination schedule to my patients because it's essential to contribute to the protection of the population (community immunity).	1-5	4.95(0.22)	20	5(0)	20
Trust in authorities	I trust the National Vaccination Programme authorities to ensure that vaccines are safe.	1-5	4.90(0.31)	20	5(0)	20
Proactive efficacy		1-5	4.43(0.24)	20	4.73(0.30)	20
	I am committed in ensuring that my patients are vaccinated.	1-5	4.95(0.22)	20	5(0)	20
	I feel sufficiently trained on how to bring up the question of vaccines with hesitant patients.	1-5	3.90(0.45)	20	4.45(0.61)	20
Openness to patients	I inform my patients about the benefits and risks of vaccines without trying to influence them.	1-5	3.95(1.10)	20	4.40(0.94)	20
Perceived constraints	The cost of some vaccines is a problem for some patients and can keep me from prescribing them.	1-5	2.60(1.54)	20	2.25(1.33)	20
Reluctant trust	I may sometimes recommend vaccines from the official schedule even if I feel the vaccination policy is not sufficiently clear.	1-5	3.42(1.43)	19	3.35(1.76)	20
Perceived difficulties to refute counter-arguments		1-5	3.91(2.43)	20	1.87(0.81)	20
	The authorities are lying and covering up important information about the vaccine.	1-5	2.95(1.32)	20	2.10(1.17)	20
	There is not enough safety testing, and no one is liable if someone is harmed by the vaccine.	1-5	2.70(1.38)	20	2.15(1.23)	20
	Scientists are still debating the benefits of vaccination, and the science is not settled.	1-5	2(0.86)	20	1.65(1.14)	20
	Politicians use vaccinations as strategies to boost their own political agendas at the expense of the common good.	1-5	2.50(1.19)	20	2.05(1.32)	20
	The human body was created in God's image, so it is a sin to defile it with unnatural injections.	1-5	2.58(1.39)	19	2.21(1.44)	19
	People should not accept vaccines that are produced using tissues from aborted foetuses.	1-5	2(1.29)	19	1.79(1.08)	19
	Vaccines overwhelm the immune system, especially when taken in many doses.	1-5	1.79(0.98)	19	1.37(0.50)	19
	Vaccinations are unnecessary if you have a strong immune system that protects you from vaccine-preventable diseases.	1-5	2.32(1.11)	19	1.68(1.06)	19
	People whose jobs allow them to adopt strong preventive measures against diseases should not need to get vaccinated.	1-5	2.21(1.03)	19	1.42(0.61)	19
	Negative experiences and testimonies of injuries by patients should be prioritised when deciding whether or not to accept vaccination.	1-5	2.95(1.18)	19	1.85(1.09)	20
	People should be able to decide what goes into their bodies, so it should be a matter of free personal choice whether someone gets a vaccine.	1-5	2.89(1.33)	19	2.05(1.32)	20

Note. Sample size not large enough to compute reliability analyses.

Supplementary Table 5

Items administered to physicians in the ERI group.

Variable	Question	Response options	Pre-test N	M(SD)	Post-test N	M(SD)
Knowledge about ERI			10	8.70(2.26)	10	13.10(1.29)
	Circle all of the 11 common attitude roots related to vaccination (pre-test reformulate					

the question “Based on your knowledge what are the 11 most common attitude roots related to vaccination?”).			
	Extroversion	0	0
	**Fear & phobias**	9	10
	Social identity	4	5
	**Conspiracist ideation**	9	10
	Neuroticism	0	0
	**Epistemic relativism**	1	9
	**Distrust**	10	9
	**Worldview & politics**	4	10
	**Perceived self-interest**	4	9
	Openness to experience	0	0
	**Religious concerns**	7	10
	**Unwarranted beliefs**	9	10
	Locus of control	1	1
	**Moral concerns**	5	8
	Ethnicity	4	3
	**Distorted risk perception**	7	10
	**Reactance**	0	6
	Crystallised intelligence	0	0
What are two key components of a refutation? (pre-test reformulate the question “Based on your knowledge what are two of the key components of a refutation”).			
	**Explain why the misconception is wrong and provide a plausible alternative.**	6	9
	Give factual evidence and check for understanding.	5	2
	Forewarn about misinformation and provide plausible facts.	5	1
	Emphasise that the misinformation is wrong and check there is no misunderstanding.	3	0
Select the usual sequential order of the Empathetic Refutational Interview (for pre-test reformulate the question: “Based on your knowledge, select the usual sequential order of the Empathetic Refutational Interview”).			
	Elicit concerns, Affirm, Provide facts, Tailored refutation	5	2
	Provide facts, Tailored refutation, Affirm, Elicit concerns	0	0
	**Elicit concerns, Affirm, Tailored refutation, Provide facts**	5	8
	Tailored refutation, Affirm, Provide fact, Elicit concerns	0	0
In this situational exercise, imagine that you are in the clinical case described below.<br><br>You are talking to Mrs Popescu, who received a letter from your GP practice inviting her daughter Maria who is 11 years old for her HPV vaccination. But Mrs Popescu has some concerns because recently someone from her Whatsapp group shared a link to research on HPV vaccine giving side effects like infertility. After reading about this, Mrs Popescu became worried about			

	putting Maria at risk by having the vaccines.<br><br>Which of the following options demonstrates an affirmation that is appropriate to Mrs Popescu's context?					
		Yes, but HPV is a very dangerous virus, so it would be best to protect Maria with vaccination.	3		0	
		Can I share more information with you about why we offer the HPV vaccine for children at this age?	1		1	
		The HPV vaccine will give Maria the best protection we can offer against serious diseases like cervical cancer.	8		4	
		**I'm pleased you're searching for information to support the best medical decisions for Maria.**	3		8	
	Which of the following refutations is best tailored to Mrs Popescu's attitude root and concerns?					
		**I can assure you that the HPV vaccine has no such side effect as infertility, and this has been proven through a long and successful period since we vaccinate with this HPV vaccine.**	3		5	
		The HPV vaccine is safe and doesn't cause infertility. There is a lot of scientific evidence to show that this is the case.	0		1	
		Vaccination is the most effective protection we have against HPV virus. Of course it can have mild side effects, but we know a lot about these and that severe reactions are 5extremely rare.	3		1	
		To an unvaccinated and HPV infected person, if the immune system fails to clear the virus from the body within two years before it causes health problems, the infection may persist and cause genital warts, precancerous lesions and malignant tumours in the oropharyngeal or anogenital area. Studies have shown it to be safe and effective. It's best not to trust completely what people say on social media.	5		3	
Confidence in using the ERI			-	-	10	9.47(0.40)
	How do you rate your confidence level to include ERI in your professional context?	1-10	-	-	10	9.60(0.51)
	To which extent is it easy for you to discuss with a patient reporting misconceptions about immunisation?	1-10	-	-	10	9.10(0.74)
	To which extent do you feel confident in affirming such a patient?	1-10	-	-	10	9.60(0.70)
	To which extent do you feel confident in offering a tailored refutation to the patient's concerns?	1-10	-	-	10	9.40(0.52)
	To which extent do you feel confident in eliciting a patient's concerns?	1-10	-	-	10	9.10(0.99)
	To which extent do you feel confident in providing factual	1-10	-	-	10	9.80(0.42)

	information in a comprehensive format to patients?					
	To which extent do you feel prepared to conduct an ERI?	1-10	-	-	10	9.70(0.48)

Note. Correct answers in bold.

Supplementary Table 6

Items administered to physicians in the MI group.

Variable	Question	Response options	Pre-test N	Pre-test M(SD)	Post-test N	Post-test M(SD)
Knowledge about MI			10	4.10(1.10)	10	7.10(1.60)
	Identify the three (3) determinant factors in personal behavior change according to motivational interviewing.					
		**Confidence in having the ability to change**	8		9	
		Peer pressure to change	0		0	
		**Importance given to change**	8		8	
		Anticipated rewards and pleasures	4		1	
		**Readiness to change**	4		7	
		Tips and suggestions of others regarding change	6		5	
	What are the essentials skills used in motivational interviewing?					
		Tips, directives, reflective listening, open-ended questions, summarizing	3		0	
		**Open-ended questions, affirmations, reflective listening, summarizing, sharing information**	6		9	
		Open-ended questions, tips, reflective listening, information	1		0	
		Open-ended questions, reflective listening, affirmations, tips	0		1	
	Which one of the following statements agrees the most with motivational interviewing principles?					
		Provide advice on immunization guidelines	3		5	
		**Encourage parents / patients to reflect even if he or she is not planning on vaccinating his or her child**	2		5	
		Talk about the risks of non-vaccination	3		0	
		Argue in favor of immunization benefits	2		1	
	Respecting the motivational interviewing spirit, which of the following best answers this statement: "There have been many negative effects of certain vaccines that have been reported. Other than a tetanus shot, if needed, I don't see a good enough reason to follow vaccination recommendations. Not even influenza vaccine"?					
		Those are however the guidelines of the Romanian immunization program	0		0	
		This prevents a disease that can be serious at your age, with your medical condition	1		1	
		**I hear that you are concerned about vaccines side effects**	0		6	

		It may be hard for you, but it is the only way to properly protect your	9		3	
	What are the 4 fundamental attitudes of the motivational interviewing spirit?					
		Expertise, tips, directives, empathy	7		2	
		Partnership, evocation, non-judgement, altruism	0		5	
		**Empathy, sincerity, enthusiasm, support**	3		3	
		Judgement, tips, dynamism, openness	0		0	
	Which of the following best answers this question and statement: “Why should my nine-year old daughter receive a vaccine against HPV? She has not even had sexual intercourse yet.”					
		Because it is recommended to receive the vaccine at this age; the vaccine is actually more effective when the person is not infected, so it is preferable to receive it before having sexual intercourse. How do you feel about this?	6		7	
		You are wondering why the vaccine is recommended at such a young age. I will give you information regarding the administration age for this vaccine and then you will understand.	1		2	
		**What do you think could be the reasons supporting the recommendation for vaccination at this age?**	0		0	
		If this vaccine is listed on the vaccine schedule, it is therefore safe and effective. You need not worry: those are the guidelines of the Romanian immunization program. The vaccine is given at the best moment for you daughter’s health; this will prevent her from becoming infected.	3		1	
Behaviors related to the MI			10	3.88(0.66)	10	4.64(0.51)
	You are a vaccinator. You receive Maria who is 27 years old. She wants to know about the HPV vaccine, but she is very anxious. “It is amazing how many people bent over and trusted untested, "medicine" like Covid vaccine. Can I trust HPV is different?		10		10	
		HPV vaccine is the most effective protection we have against the HPV virus.	7		6	
		**I understand that you have questions about how the HPV vaccine was tested.**	3		6	
		HPV is a very dangerous virus, so you can be confident that vaccination is the best solution in this case.	1		0	
		It is not at all true that the COVID vaccine has not been tested.	3		0	
		**I am glad you are looking for information to make the best decision for your health.**	4		9	

		In fact, researchers have extensively studied and tested this vaccine.	2		0	
		**Can I share more information about vaccine testing to help clarify this subject that is worrying you?**	6		7	
	I provide information to parents or patients straight away, without them having to ask for it.	1-6	10	1.30(0.48)	10	1.50(0.71)
	I identify with the parents/patients the best choice for them while discussing immunization.	1-6	10	5.20(0.79)	10	5.70(0.68)
	I acknowledge the parent's or patient's efforts to reach out for information on immunization on their own.	1-6	10	3.90(1.79)	10	5.20(0.92)
	If discussed, I summarize the benefits and disadvantages identified by parents /patients regarding immunization.	1-6	10	5.50(0.79)	10	5.30(0.68)
	I attempt straight away to convince parents of the importance of vaccinating their child.	1-6	10	1.40(0.70)	9	2.78(2.05)
	I begin with what they know about vaccines as a starting point for our conversation.	1-6	10	4.80(1.23)	10	5.30(0.95)
	I encourage parents by reminding them that the decision is theirs and that I am confident they will make the right choice for their child.	1-6	10	3.90(1.85)	10	5.60(0.70)
	I try to understand how parents perceive their child's vaccination in their desire not to vaccinate.	1-6	10	4.40(1.51)	10	5.30(0.95)
	I tell parents that if decision were mine, I would vaccinate my child.	1-6	10	5.40(1.58)	9	4.89(1.45
	I invite parents /patients to give me feedback on the immunization information that I provided.	1-6	10	4.20(1.55)	10	4.60(1.65)
	I reflect the emotions experienced by the parents / patients.	1-6	10	3.30(1.49)	10	4.40(1.51)
	I try to understand the arguments of parents /patients hesitant to immunization without correct them straight away.	1-6	10	3.30(1.77)	10	4.80(1.03)
Confidence in using the MI		1-10	-	-	9	7.94(1.70)
	How do you rate your confidence level to pursue motivational interviewing practice in your professional context?	1-10	-	-	9	8.56(1.24)
	To which extent do you feel comfortable in initiating a conversation about change with your clients?	1-10	-	-	9	7.44(2.70)
	To which extent is it easy for you to discuss with a client mostly closed-minded to certain vaccines or to immunization in general?	1-10	-	-	8	8.38(1.41)
	To which extent do you feel confident in listening with empathy to an open- or closed-minded to immunization client?	1-10	-	-	8	8.50(1.41)
	To which extent do you feel confident in reflecting the reality experienced by parents?	1-10	-	-	8	8.63(1.41)
	To which extent do you feel prepared to conduct a motivational interview?	1-10	-	-	8	8.13(1.81)

Note. Correct answers in bold.

Supplementary Table 7

Items administered to the patients.

			Pre-test			Post-test		
Variable	Item	Range	M(SD)	α	N	M(SD)	α	N
7C of vaccination readiness scale		1-7	4.70(1.39)	0.88	334	5.54(1.17)	0.82	334
	I am convinced the appropriate authorities do only allow effective and safe vaccines.	1-7	5.18(1.62)		334	6.06(1.29)		334
	I get vaccinated because it is too risky to get infected.	1-7	5.10(1.69)		334	6.12(1.28)		333
	Vaccinations are so important to me that I prioritize getting vaccinated over other things.	1-7	4.50(1.76)		334	5.59(1.49)		334
	I only get vaccinated when the benefits clearly outweigh the risks. (R)	1-7	5.21(1.77)		329	5.89(1.65)		333
	I see vaccination as a collective task against the spread of diseases.	1-7	4.89(1.88)		333	5.84(1.57)		333
	It should be possible to sanction people who do not follow the vaccination recommendations by health authorities.	1-7	3.09(2.14)		332	3.73(2.30)		333
	Vaccinations cause diseases and allergies that are more serious than the diseases they ought to protect from. (R)	1-7	4.94(1.95)		333	5.57(1.93)		334
Willingness to vaccinate	Please state how willing are you to receive the vaccines that are recommended for you.	1-7	4.78(1.82)		330	5.96(1.57)		330
Appointment	Have you received, or scheduled an appointment to receive, the recommended vaccine after the consultation?	2				1.69(0.46)		317
Satisfaction		1-5				4.32(0.55)	0.81	333
	Did you enjoy participating in this interview?	1-5				4.56(0.76)		330
	Would you recommend that this type of interview be offered to other patients during a medical consultation?	1-5				4.64(0.73)		327
	Do you feel that the interview respected your views on vaccination?	1-5				4.77(0.54)		327
	Did you find it useful to discuss vaccination with the provider?	1-5				4.67(0.73)		332
	Was the length of the interview...	1-5				2.94(0.65		314
Doubts	Following this interview, do you still have questions about vaccination?	2				1.11(0.32)		227

Note: Means, standard deviations, and Cronbach's alphas of the total sample. (R) = Reversed encoded.
